# Clinical and Genetic Characterization of a Novel RYR1 Variant (p.Gln474His) in Malignant Hyperthermia Susceptibility

**DOI:** 10.3390/genes17010013

**Published:** 2025-12-24

**Authors:** Erin Tracy, Katelyn Mistretta, Peter Bedocs, Robert Vietor, Alakesh Bera

**Affiliations:** Department of Anesthesiology, School of Medicine, Uniformed Services University of the Health Sciences, 4301 Jones Bridge Rd., Bethesda, MD 20814, USA

**Keywords:** *RYR1* variant, malignant hyperthermia, and caffeine-halothane contracture test (CHCT)

## Abstract

Background/Objectives: Malignant hyperthermia (MH) is a life-threatening pharmacogenetic disorder of skeletal muscle calcium regulation and commonly associated with pathogenic variants in the RYR1 gene. Interpretation of rare RYR1 variants remains challenging, particularly when classified as variants of uncertain significance (VUS). This study describes the clinical, functional, and genetic evaluation of a patient with suspected MH susceptibility carrying a rare RYR1 mutation. Methods: We report a retrospective case evaluation of a 32-year-old female referred for MH assessment following a prior peri-operative hypermetabolic event. Clinical records were reviewed, and MH susceptibility was assessed using the caffeine–halothane contracture test (CHCT). Genetic testing was performed using a targeted MH susceptibility gene panel, including RYR1, CACNA1S, and STAC3. Variant classification was conducted following American College of Medical Genetics and Genomics/Association for Molecular Pathology (ACMG/AMP) guidelines. Results: The patient demonstrated a positive CHCT, consistent with MH susceptibility. Genetic analysis identified a rare heterozygous RYR1 missense variant. No pathogenic or likely pathogenic variants were detected in CACNA1S or STAC3. Based on ACMG/AMP criteria, the RYR1 p.Gln474His variant is currently classified as a VUS. However, its localization within the N-terminal regulatory region of RyR1 and concordance with abnormal CHCT findings provide supportive functional context. Conclusions: This case underscores the importance of integrating clinical history, functional contracture testing, and genetic data in the evaluation of MH susceptibility. While functional findings may support biological plausibility, definitive pathogenic classification of rare RYR1 variants requires additional segregation data or mechanistic studies.

## 1. Introduction

Malignant hyperthermia (MH) is a life-threatening pharmacogenetic disorder of skeletal muscle calcium (Ca^2+^) regulation. It is classically triggered by volatile anesthetics or the depolarizing muscle relaxant succinylcholine, leading to uncontrolled Ca^2+^ release from the sarcoplasmic reticulum, sustained muscle contraction, and a hypermetabolic crisis [1,2,3,4,5,6,7]. Epidemiologic estimates suggest that MH occurs in approximately 1 in 5000–50,000 anesthetic exposures, while the underlying genetic susceptibility is far more common and it is approximately 1 in 1000 individuals harbor a potentially MH-related variant [8,9]. Clinically, this manifests as hypercapnia (rapid rise in end-tidal CO_2_), tachycardia, increased oxygen consumption, hyperthermia, acidosis, rhabdomyolysis, and hyperkalemia. The absence of treatment may lead to multisystem organ failure and death [6,10,11]. The skeletal muscle ryanodine receptor (RyR1), encoded by the *RYR1* gene, is the principal Ca^2+^ release channel responsible for excitation–contraction coupling. The gain-of-function RYR1 mutations account for approximately 50–70% of MH susceptibility cases [12,13]. Variants in *CACNA1S*, which encodes the α1s subunit of the dihydropyridine receptor (DHPR), explain about 1% of MH cases, while rare variants in STAC3 have also been implicated [2,14,15,16,17]. It is also documented that positive CHCT test results have been observed in X-linked muscle dystrophies such as patients with Duchenne muscular dystrophy (DMD) and with Becker muscular dystrophy (BMD) [18].

The in vitro contracture test (IVCT), or caffeine–halothane contracture test (CHCT), remains the gold standard for functional diagnosis of MH susceptibility (MHS) [4,7,19,20]. The test measures isometric muscle contracture in response to halothane and caffeine, with established thresholds (e.g., ≥0.4–0.5 g with 2% halothane; ≥0.3 g with 2 mM caffeine) defining MHS [21,22,23]. While genetic testing is increasingly integrated into MH evaluation, it cannot fully replace IVCT because of genetic heterogeneity and the high prevalence of variants of uncertain significance (VUS). The interpretation of novel or rare RYR1 variants represents a major challenge. Many are reported as VUS due to insufficient functional or segregation data, creating uncertainty for patient management and anesthetic planning. To standardize interpretation, the American College of Medical Genetics and Genomics (ACMG) provides evidence-based guidelines that integrate population frequency, computational prediction, segregation data, and functional assays [9,24,25]. In addition to its well-established clinical presentation, accurate assessment of malignant hyperthermia susceptibility increasingly relies on a combination of clinical history, in vitro functional testing, and molecular genetic data, each of which contributes complementary evidence toward variant interpretation. However, full pathogenic classification still requires segregation analysis or mechanistic confirmation, underscoring the importance of cautious interpretation when evaluating newly identified RYR1 variants.

Here, we present the case of a 32-year-old female who developed intraoperative signs concerning for MH during an appendectomy. Her episode resolved after discontinuation of volatile anesthesia and conversion to total intravenous anesthesia (TIVA). Genetic testing using the Invitae MH susceptibility panel (*RYR1*, *CACNA1S*, *STAC3*) revealed a heterozygous *RYR1* c.1422G>C (p.Gln474His) variant, currently classified as a VUS. By documenting the clinical phenotype, genetic findings, and diagnostic uncertainty in this case, we aim to contribute to the evidence base for *RYR1* c.1422G>C (p.Gln474His), highlight the limitations of current testing strategies, and underscore the importance of functional validation and case-based reporting in reclassifying VUS to improve MH risk stratification and patient safety.

## 2. Case Presentation

A 32-year-old Hispanic female (IRB# ANE-08-3397; Uniformed Services University; approval date: 14 April 2025, valid until 1 April 2026) was referred to our Malignant Hyperthermia (MH) program for evaluation of a suspected prior MH episode. Her index event occurred at age 16 during an emergency open appendectomy. According to the operative and anesthesia records which are obtained later as part of her clinical care. Records indicated that she was induced with midazolam 2 mg, fentanyl 150 µg, propofol 140 mg, and succinylcholine 100 mg, followed by rocuronium 20 mg after confirming neuromuscular recovery. Anesthesia was maintained with desflurane in oxygen.

Approximately halfway into the procedure, she developed gradually worsening tachycardia (HR 130→160 bpm), a rise in end-tidal CO_2_ from the 40 s (PaCO_2_ 41 mmHg) to the high 60 s mmHg despite increased ventilation, and a temperature increase from 36.0 °C to 38.3 °C. No masseter spasm or generalized rigidity was noted. After concern for MH arose, desflurane was discontinued and total intravenous anesthesia (TIVA) with propofol was initiated. Arterial blood gas analysis showed pH 7.32, CO_2_ 41 mmHg, lactate 5.2 mmol/L, and bicarbonate 20 mEq/L. The patient was extubated uneventfully.

Postoperatively, her serum creatine kinase (CK) was 7000 U/L in the PACU and rose to 17,000 U/L on postoperative day 2. CK-MB and troponin levels were transiently elevated, and a 12-lead ECG showed acute pericarditis with inferior Q waves; echocardiography demonstrated normal cardiac function. She recovered without Dantrolene administration. Dantrolene is the only specific pharmacologic antidote for malignant hyperthermia. It acts by inhibiting excessive calcium release from the sarcoplasmic reticulum through the ryanodine receptor (RYR1), thereby reversing the hypermetabolic crisis and preventing life-threatening complications. These findings were clinically concerning towards malignant hyperthermia.

Years later (in 2024), the patient presented to Walter Reed National Military Medical Center for evaluation of persistent musculoskeletal symptoms. At that time, her prior anesthesia records and clinical history were obtained solely as part of routine care, and not as research data. After clinical evaluation, she was invited to participate in our IRB-approved MH research protocol. Upon obtaining informed consent, and under continuous annual IRB approval, she underwent study-related procedures including peripheral blood collection, muscle biopsy, and caffeine–halothane contracture testing (CHCT). Genetic testing for *RYR1* variants was subsequently performed as part of the research protocol.

## 3. Genetic Results

**Figure 1:**
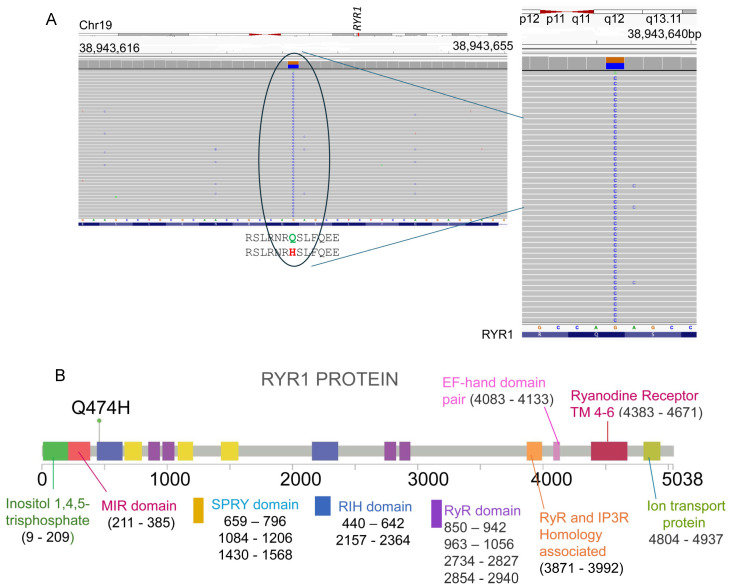
(**A**). Next-generation sequencing alignment showing the RYR1 c.1422G>C variant (p.Q474H). Integrated Genomics Viewer (IGV) alignment illustrates the heterozygous nucleotide substitution at chromosome 19 (chr19: 38,943,640 bp region), where a reference G is replaced by C, resulting in the amino acid change glutamine (Q) to histidine (H) at position 474. (**B**). Domain-level mapping of the RYR1 Q474H variant within the N-terminal region of the protein. Schematic representation of the full-length RYR1 protein depicting major functional domains, including MIR, RIH, SPRY, RyR domains, and transmembrane segments. The Q474H mutation is highlighted within the N-terminal (RIH) domain. The N-terminal region is critical for channel gating and intramolecular regulation, and is a recognized hotspot for disease-associated variants.

Genetic analysis was performed using the Invitae Malignant Hyperthermia Susceptibility Panel (Invitae part of LabCorp Genetics, San Francisco, CA, 94103). Sequencing included *RYR1* (NM_000540.2), *CACNA1S* (NM_000069.2), and *STAC3* (NM_145064.2). A heterozygous missense variant was identified in RYR1 (c.1422G>C; p.Gln474His) (Figure 1). No pathogenic variants were found in CACNA1S or STAC3 (Table 1).

## 4. Results and Discussion

Malignant hyperthermia (MH) is a pharmacogenetic disorder of skeletal muscle calcium regulation, most often triggered by volatile anesthetics or succinylcholine, and characterized by hypercapnia, tachycardia, acidosis, hyperthermia, and rhabdomyolysis [6,8,21,26]. Variants in the RYR1 gene, which encodes the principal sarcoplasmic reticulum calcium release channel, account for the majority (~75%) of MH susceptibility (MHS) cases [9,26,27,28].

In this report, we describe a 32-year-old female carrying a novel heterozygous missense variant in *RYR1* (c.1422G>C; p.Gln474His). This variant is absent from population databases, supporting its rarity. The patient experienced an intraoperative crisis at age 16 during an emergency appendectomy, showing hallmark MH features including tachycardia (130–160 bpm), elevated end-tidal CO_2_ (rising from the 40 s to 60 s mmHg despite hyperventilation), and hyperthermia (36.0–38.3 °C). Laboratory findings revealed a striking postoperative serum creatine kinase (CK) peak of 17,000 U/L (normal < 200 U/L), indicating severe rhabdomyolysis, accompanied by transient troponin elevation and acute pericarditis. Together, these findings represent a strong clinical correlation with MH [29].

**Figure 2:**
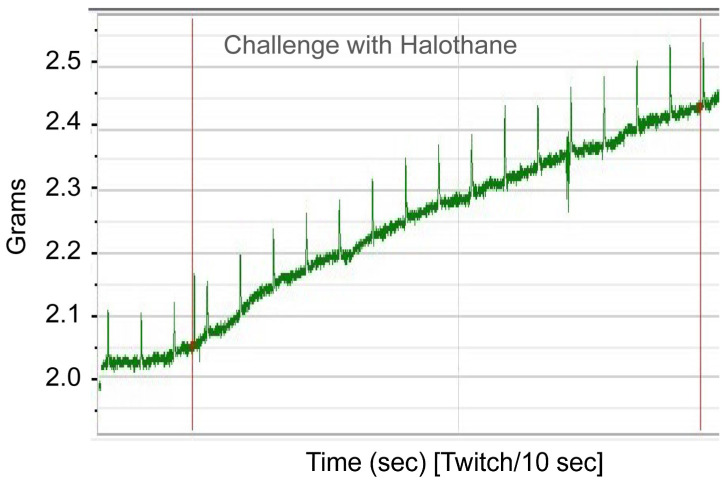
Caffeine–Halothane Contracture Test (CHCT) screening using halothane challenge. Skeletal muscle specimen was mounted in an in vitro bath chamber containing Krebs–Henseleit solution, continuously oxygenated with ~95% O_2_/5% CO_2_ to maintain physiological conditions. Muscle contracture response was measured following exposure to halothane. The diagnostic halothane concentration used in this step was 3% (*v*/*v*, bubbled into the Krebs solution), which represents the standardized threshold for assessing malignant hyperthermia (MH) susceptibility. The red vertical lines indicate the time window during which the most significant contractile responses were observed following challenge with halothane (Figure 2) or caffeine (Figure 3).

**Figure 3:**
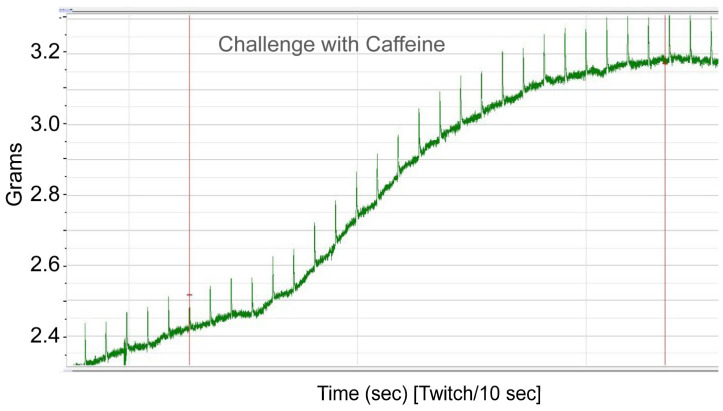
Caffeine–Halothane Contracture Test (CHCT) screening using caffeine challenge. Skeletal muscle specimen was exposed to increasing concentrations of caffeine in Krebs–Henseleit solution, continuously oxygenated with ~95% O_2_/5% CO_2_. The caffeine concentration was stepwise increased from 0.5 mM to 4.0 mM to assess contracture development. A diagnostic contracture response was observed at 1.5 mM caffeine, indicating abnormal calcium release consistent with malignant hyperthermia (MH) susceptibility.

To functionally confirm MH susceptibility, the patient underwent the caffeine–halothane contracture test (CHCT), which is the gold standard diagnostic test (Figure 2 and Figure 3). Her muscle strips showed significant contractures in response to both halothane and caffeine, fulfilling diagnostic thresholds and confirming MHS (Figure 2 and Figure 3; Table 2). CHCT contracture responses were interpreted using established North American diagnostic threshold values, rather than direct comparison with normal/healthy control muscle specimens. All halothane and caffeine challenge experiments were performed in triplicate. This result provides direct functional evidence of dysregulated calcium release, the central defect in MH.

According to the American College of Medical Genetics and Genomics/Association for Molecular Pathology (ACMG/AMP) framework, the *RYR1* p.Gln474His variant is currently classified as a Variant of Uncertain Significance (VUS) [25,30]. However, several ACMG criteria support consideration for reclassification. Specifically, the strong functional evidence from the CHCT (PS3) and the highly specific clinical phenotype consistent with MH (PP4) suggest that this variant may warrant classification as “Likely Pathogenic (Table 3).” The presence of peri-operative crisis, markedly elevated CK, and associated cardiac complications further strengthen this interpretation.

Notably, the p.Gln474His variant lies within the N-terminal region of RyR1, a recognized mutational hotspot associated with both MH and congenital myopathies, including central core disease (CCD) disease [20,31,32,33]. This N-terminal domain including RIH (RyR–IP_3_ receptor homology) domain contributes to formation of the cytoplasmic vestibule and plays a critical role in stabilizing closed-state channel gating. Disease-associated variants in the N-terminal domains have been shown to disrupt intramolecular interactions, lowering the activation threshold and predisposing the channel to pathological calcium leak. Localization of p.Gln474His within this functionally important region therefore provides biological plausibility for its role in MH susceptibility. Previous studies have shown that disease-associated variants in this region alter the cytoplasmic vestibule of the channel, destabilizing closed-state regulation and predisposing to pathological calcium leaks. The localization of this variant within such a hotspot adds further biological plausibility to its pathogenicity. An unusual feature in this case is the presence of cardiac sequelae, namely transient troponin elevation and acute pericarditis, following the MH-like crisis. While RYR1-related congenital myopathies typically do not involve the heart, this observation raises the possibility that certain RYR1 variants could predispose to extra-skeletal manifestations under metabolic stress. This highlights the importance of multidisciplinary follow-up and ongoing cardiac surveillance in MH-susceptible patients.

Despite compelling clinical and functional evidence, definitive pathogenicity assignment for p.Gln474His requires additional studies. Although two independent submissions reporting the same RYR1 variant (p.Gln474His, Q474H) are present in ClinVar, including one expert-reviewed submission, the variant is currently classified as a variant of uncertain significance (VUS). Segregation analysis in family members would clarify whether the variant tracks with susceptibility. Furthermore, in vitro calcium release assays using heterologous expression systems could provide mechanistic insight into its effect on channel gating and calcium sensitivity. These investigations, combined with case reports like ours, are essential for variant reclassification, enriching MH variant databases, and guiding patient management.

## 5. Conclusions

We report a 32-year-old female with malignant hyperthermia susceptibility confirmed by CHCT and associated with a novel *RYR1* variant (c.1422G>C; p.Gln474His). Although currently a VUS, integration of genetic, clinical, and functional data suggests a likely pathogenic role. This case adds to the growing body of literature on RYR1 variants and highlights the importance of comprehensive assessment for proper risk stratification and patient safety.

Although the phenotypic findings and CHCT results support a potential role for the RYR1 c.1422G>C (p.Gln474His) variant in malignant hyperthermia susceptibility, we acknowledge that this interpretation remains suggestive rather than definitive. Reclassification toward ‘likely pathogenic’ cannot be made without additional evidence such as segregation analysis in affected family members or functional validation beyond CHCT, including protein-level or cellular assays. Any potential systemic implications including theoretical cardiac involvement are presented here as speculative considerations and not as established clinical associations. These hypotheses are offered to guide future investigation and should not be interpreted as conclusive evidence.

## Figures and Tables

**Table 1:** Genetic test data and Variant details for MH related genes. VUS = variants of uncertain significance. Negative = not known any pathogenesis.

Gene	mRNA Transcript	Nucleotide Change	Protein Change	Classification
*RYR1*	NM_000540.2	c.1422G>C	p.Gln474His	VUS
*CACNA1S*	NM_000069.2	No variant	-	Negative
*STAC3*	NM_145064.2	No variant	-	Negative

**Table 2:** CHCT test results.

Test	Halothane Challenge (1–3%)	Caffeine Challenge (0.5–4 mM)	Result	Interpretation
Caffeine–Halothane Contracture Test (CHCT)	Max. Contracture at 2.5–3%	Max. Contracture at 1.5 mM	Positive	Confirmed MH Susceptible (MHS)

**Table 3:** ACMG Classification Evidence for the identified RYR1 variant, evaluated using ACMG/AMP 2015 guidelines with integrated clinical, genetic, functional, and in silico data to determine final pathogenicity [25]. Abbreviation: PM = Pathogenic Moderate; PP = Pathogenic Supporting; PS = Pathogenic Strong.

Criteria	Strength	Evidence
PM2	Moderate	Absent from large population databases (gnomAD).
PP3	Supporting	Computational predictions suggest a deleterious effect.
PS3	Strong	Functional evidence: positive CHCT confirming abnormal calcium handling.
PP4	Supporting	Clinical presentation specific for MH (peri-operative crisis with CK elevation).

## Data Availability

All original data supporting the findings of this study are included in the article. Additional information is available from the corresponding authors upon reasonable request.

## References

[1] Brady J.E., Sun L.S., Rosenberg H., Li G. (2009). Prevalence of malignant hyperthermia due to anesthesia in New York State, 2001–2005. Anesth. Analg..

[2] Fiszer D., Shaw M.A., Fisher N.A., Carr I.M., Gupta P.K., Watkins E.J., Roiz de Sa D., Kim J.H., Hopkins P.M. (2015). Next-generation Sequencing of RYR1 and CACNA1S in Malignant Hyperthermia and Exertional Heat Illness. Anesthesiology.

[3] Monnier N., Krivosic-Horber R., Payen J.F., Kozak-Ribbens G., Nivoche Y., Adnet P., Reyford H., Lunardi J. (2002). Presence of two different genetic traits in malignant hyperthermia families: Implication for genetic analysis, diagnosis, and incidence of malignant hyperthermia susceptibility. Anesthesiology.

[4] Ording H., Brancadoro V., Cozzolino S., Ellis F.R., Glauber V., Gonano E.F., Halsall P.J., Hartung E., Heffron J.J., Heytens L. (1997). In vitro contracture test for diagnosis of malignant hyperthermia following the protocol of the European MH Group: Results of testing patients surviving fulminant MH and unrelated low-risk subjects. The European Malignant Hyperthermia Group. Acta Anaesthesiol. Scand..

[5] Rosenberg H., Fletcher J.E. (1986). Masseter muscle rigidity and malignant hyperthermia susceptibility. Anesth. Analg..

[6] Rosenberg H., Pollock N., Schiemann A., Bulger T., Stowell K. (2015). Malignant hyperthermia: A review. Orphanet J. Rare Dis..

[7] Rosenberg H., Reed S. (1983). In vitro contracture tests for susceptibility to malignant hyperthermia. Anesth. Analg..

[8] Hopkins P.M., Gupta P.K., Bilmen J.G. (2018). Malignant hyperthermia. Handb. Clin. Neurol..

[9] Johnston J.J., Dirksen R.T., Girard T., Hopkins P.M., Kraeva N., Ognoon M., Radenbaugh K.B., Riazi S., Robinson R.L., Saddic L.A. (2022). Updated variant curation expert panel criteria and pathogenicity classifications for 251 variants for RYR1-related malignant hyperthermia susceptibility. Hum. Mol. Genet..

[10] Wang Q.L., Fang Y., Jin S.G., Liang J.T., Ren Y.F. (2022). Atypical symptoms of malignant hyperthermia: A rare causative mutation in the RYR1 gene. Open Med..

[11] Weiss R.G., O’Connell K.M., Flucher B.E., Allen P.D., Grabner M., Dirksen R.T. (2004). Functional analysis of the R1086H malignant hyperthermia mutation in the DHPR reveals an unexpected influence of the III-IV loop on skeletal muscle EC coupling. Am. J. Physiol. Cell Physiol..

[12] Lopez R.J., Byrne S., Vukcevic M., Sekulic-Jablanovic M., Xu L., Brink M., Alamelu J., Voermans N., Snoeck M., Clement E. (2016). An RYR1 mutation associated with malignant hyperthermia is also associated with bleeding abnormalities. Sci. Signal..

[13] van den Bersselaar L.R., van Alfen N., Kruijt N., Kamsteeg E.J., Fernandez-Garcia M.A., Treves S., Riazi S., Yang C.Y., Malagon I., van Eijk L.T. (2023). Muscle Ultrasound Abnormalities in Individuals with RYR1-Related Malignant Hyperthermia Susceptibility. J. Neuromuscul. Dis..

[14] Silva M.S., Nakamura R., Arjona M.R., Monaco T., Malito M.L., Sampaio T.O., Oliveira S.L., Magalhaes J.S.A., Machado-Costa M.C., Silva H.C.A. (2025). STAC3 gene congenital myopathy and malignant hyperthermia: A crossroads between neurology and anesthesia. Arq. Neuropsiquiatr..

[15] Zaharieva I.T., Sarkozy A., Munot P., Manzur A., O’Grady G., Rendu J., Malfatti E., Amthor H., Servais L., Urtizberea J.A. (2018). STAC3 variants cause a congenital myopathy with distinctive dysmorphic features and malignant hyperthermia susceptibility. Hum. Mutat..

[16] Roberts D.A., Bastarache L., He J., Lewis A., Aka I.T., Shotwell M.S., Reddy S.K., Hogan K.J., Biesecker L.G., Kertai M.D. (2025). Updating probability of pathogenicity for RYR1 and CACNA1S exon variants in individuals without malignant hyperthermia after exposure to triggering anesthetics. Pharmacogenetics Genom..

[17] Stewart S.L., Hogan K., Rosenberg H., Fletcher J.E. (2001). Identification of the Arg1086His mutation in the alpha subunit of the voltage-dependent calcium channel (CACNA1S) in a North American family with malignant hyperthermia. Clin. Genet..

[18] Heiman-Patterson T.D., Natter H.M., Rosenberg H.R., Fletcher J.E., Tahmoush A.J. (1986). Malignant hyperthermia susceptibility in X-linked muscle dystrophies. Pediatr. Neurol..

[19] Clarke J., Ali E., Hopkins P.M. (2020). Do in vitro pharmacological challenge responses differ between muscle specimens from malignant hyperthermia probands and their susceptible relatives?. Br. J. Anaesth..

[20] Robinson R.L., Brooks C., Brown S.L., Ellis F.R., Halsall P.J., Quinnell R.J., Shaw M.A., Hopkins P.M. (2002). RYR1 mutations causing central core disease are associated with more severe malignant hyperthermia in vitro contracture test phenotypes. Hum. Mutat..

[21] Fiege M., Wappler F., Weisshorn R., Ulrich Gerbershagen M., Steinfath M., Schulte Am Esch J. (2002). Results of contracture tests with halothane, caffeine, and ryanodine depend on different malignant hyperthermia-associated ryanodine receptor gene mutations. Anesthesiology.

[22] Gerbershagen M.U., Wappler F., Fiege M., Weilsshorn R., Alberts P.A., von Breunig F., Schulte am Esch J. (2002). In vitro effects of 4-chloro-3-ethylphenol in skeletal muscle preparations from malignant hyperthermia susceptible and normal swine. Eur. J. Anaesthesiol..

[23] Hopkins P.M., Hartung E., Wappler F. (1998). Multicentre evaluation of ryanodine contracture testing in malignant hyperthermia. The European Malignant Hyperthermia Group. Br. J. Anaesth..

[24] Li M.M., Datto M., Duncavage E.J., Kulkarni S., Lindeman N.I., Roy S., Tsimberidou A.M., Vnencak-Jones C.L., Wolff D.J., Younes A. (2017). Standards and Guidelines for the Interpretation and Reporting of Sequence Variants in Cancer: A Joint Consensus Recommendation of the Association for Molecular Pathology, American Society of Clinical Oncology, and College of American Pathologists. J. Mol. Diagn..

[25] Richards S., Aziz N., Bale S., Bick D., Das S., Gastier-Foster J., Grody W.W., Hegde M., Lyon E., Spector E. (2015). Standards and guidelines for the interpretation of sequence variants: A joint consensus recommendation of the American College of Medical Genetics and Genomics and the Association for Molecular Pathology. Genet. Med..

[26] Hopkins P.M. (2023). What is malignant hyperthermia susceptibility?. Br. J. Anaesth..

[27] Kim K., Li H., Yuan Q., Melville Z., Zalk R., des Georges A., Frank J., Hendrickson W.A., Marks A.R., Clarke O.B. (2024). Structural identification of the RY12 domain of RyR1 as an ADP sensor and the target of the malignant hyperthermia therapeutic dantrolene. bioRxiv.

[28] Levano S., Vukcevic M., Singer M., Matter A., Treves S., Urwyler A., Girard T. (2009). Increasing the number of diagnostic mutations in malignant hyperthermia. Hum. Mutat..

[29] Andrade P.V., Santos J.M., Teixeira A.C.B., Sogari V.F., Almeida M.S., Callegari F.M., Krepischi A.C.V., Oliveira A.S.B., Vainzof M., Silva H.C.A. (2023). Rhabdomyosarcoma Associated with Core Myopathy/Malignant Hyperthermia: Combined Effect of Germline Variants in RYR1 and ASPSCR1 May Play a Role. Genes.

[30] Green R.C., Berg J.S., Grody W.W., Kalia S.S., Korf B.R., Martin C.L., McGuire A.L., Nussbaum R.L., O’Daniel J.M., Ormond K.E. (2013). ACMG recommendations for reporting of incidental findings in clinical exome and genome sequencing. Genet. Med..

[31] Broman M., Islander G., Muller C.R., Ranklev-Twetman E. (2007). Malignant hyperthermia and central core disease causative mutations in Swedish patients. Acta Anaesthesiol. Scand..

[32] Chen W., Koop A., Liu Y., Guo W., Wei J., Wang R., MacLennan D.H., Dirksen R.T., Chen S.R.W. (2017). Reduced threshold for store overload-induced Ca(2+) release is a common defect of RyR1 mutations associated with malignant hyperthermia and central core disease. Biochem. J..

[33] Robinson R., Carpenter D., Shaw M.A., Halsall J., Hopkins P. (2006). Mutations in RYR1 in malignant hyperthermia and central core disease. Hum. Mutat..

